# The Hospital. Nursing Section

**Published:** 1904-05-28

**Authors:** 


### The Hospital.

### Nursing Section.

Contributions for this Section of "The Hospital" should be addressed to the Editor, "The Hospital" Nursing Section, 28 & 29 Southampton Street, Strand, London, W.C.

No. 922.—Vol. XXXVI. *SATURDAY, MAY* 28, 1904.

### Notes on News from the Nursing World.

### QUEEN ALEXANDRA'S NAVAL NURSING SERVICE.

We understand that by an order dated May 18th the following sisters have been confirmed in their appointments in Queen Alexandra's Royal Naval Nursing Service:—Miss Annie Stanton, Miss Ada E. Woodruff, Miss Maud M. Phelan, Miss Eleanor Seale, Miss Florence Bates, of the Royal Naval Hospital, Haslar; and Miss Elizabeth Grattan, of the Royal Naval Hospital, Chatham.

### THE QUEEN AND DISTRICT NURSING.

The excellent work carried on by the King's Lynn Nursing Association has been recognised by the Queen in a very pleasant manner. Her Majesty has sent a donation of £10 to the funds. Naturally she takes a special interest in an organisation so close to the doors of Sandringham, which is affiliated to Queen Victoria's Jubilee Nurses' Institute, but her gracious contribution is characteristic of her sympathy with the efforts of the district nurse in all parts of the country.

### THE PENSION FUND AND ANCOATS HOSPITAL.

On Wednesday evening last week a meeting was held in the out-patient hall of Ancoats Hospital, Manchester, and no fewer than 200 nurses assembled to hear an address from Mr. Louis Dick, the secretary of the Royal National Pension Fund, on the merits of that organisation. Mr. Councillor Royle, deputy Lord Mayor, took the chair, and in his speech advised the nurses to make provision for the future by joining the Fund. At the close of the meeting tea was provided by Mr. Helm, chairman of the hospital, and dispensed by Miss L. Chambers, the matron, in the nurses' pleasant sitting-room.

### NEW HOME FOR NURSES AT STOKE-ON-TRENT.

Last week the new King Edward the Seventh Home for Nurses in connection with the North Staffordshire Infirmary, Stoke-on-Trent, was opened by the Bishop of Shrewsbury in the presence of a large company. The foundation stone was laid by the King himself, when he was Prince of Wales, and the Home, which is a very handsome building erected at a cost of between £9,000 and £10,000, is most delightfully situated. At the opening ceremony the chairman of the infirmary board made the gratifying announcement that it is entirely free of debt.

### THE NATIONAL FUND FOR NURSES IN IRELAND.

The first report and rules of "King Edward the Seventh's Coronation National Fund for Nurses in Ireland" has been sent to us with a request that we would notice it. Perhaps the most striking feature is the exceedingly small amount of capital of a society with such an imposing title. From the account of the origin and history of the Fund we learn that the capital now consists of £4,310 Midland Railway of England debenture stock, held in the names of the three trustees, which was purchased for £3,434 15s. 6d. According to rule 27, the society is to be supported by donations, subscriptions from friends, and from the nurses themselves, interest on capital, legacies, etc. At present we understand that there are about 150 members, and it is obvious that unless the number is very largely augmented, the organisation must remain a charity, though we gather that this is not at all desired by the council of management. For our part, we think that as a charity this Fund would fulfil the most useful mission open to it. The Royal National Pension Fund already provides the greatest possible advantages which nurses can secure on a co-operative and business basis. If the Irish Fund were administered through the Royal National Fund, for the benefit of Irish nurses only, economy and financial prosperity would be secured in a manner that is impossible under existing conditions.

### THE NURSING OF CHILDREN.

The lady superintendent of the East London Hospital for Children, in an interview with our Commissioner, says she is afraid that some of the general hospitals object to receive probationers who have had special training in other institutions. The argument against it is, she thinks, that the health of young women who have been in training for three years is not likely to be so satisfactory as that of those who are fresh from home. If, however, as is the case with nurses at the East London Hospital for children, a good long rest is taken after training, the experience gained of work in the wards should be a substantial advantage. There is, perhaps, more force in the plea that this experience may tend to make them discontented with a system which, in many respects, is dissimilar from that to which they have been accustomed. At the same time, there can be no doubt of the great importance of being able to nurse children, whether the knowledge be obtained in a children's hospital or in the wards of a general hospital to which children are admitted.

### THE RELIGIOUS QUESTION AT THE NEW CARDIFF HOSPITAL.

The late Marquis of Bute promised a donation of £10,000 to the cost of the new Seamen's Hospital at Cardiff, and his will provides that this sum shall be raised to £20,000, in the event of the further amount being required in order to complete the work. In the codicil to the will signifying the enlargement of the donation, there is the following passage: "And I further direct and request my trustees, upon the said hospital being completed, to arrange that the sick received in the building shall be served by sisters of some Roman Catholic religious Order, if such arrangement can be made." The question raised in the codicil came before the General Purposes Committee of the hospital last week, and after considerable discussion a committee was appointed to confer with the trustees in London.

### EXAMINATIONS OF POOR-LAW INFIRMARY NURSES.

It is gratifying to learn that the results of the examinations of nurses of some of the principal Poor-law infirmaries are highly satisfactory. Dr. J. H. Bryant, of Guy's Hospital, who examined the probationers at Bethnal Green Infirmary, reports that out of 32 no fewer than 29 obtained the necessary number of marks to pass. Three probationers gained 75 per cent. of the possible number, and eighteen 50 per cent. Dr. Bryant states that from observations made during the oral examination, he has come to the conclusion that they have been carefully and intelligently taught, and have obtained a clear and practical knowledge of their work. The training at Camberwell Infirmary has also been pronounced by Dr. Bryant to be in every respect excellent. At Camberwell all the nine probationers obtained the requisite number of marks, and a gold medal, given by Dr. Keats, the medical superintendent, for nursing ability, gentleness, and general efficiency, was won by Miss Annette May Hawkins. Mr. Raymond Johnson, F.R.C.S., was the examiner at Highgate Hill Infirmary, and he reports that every one of the nineteen probationers who presented themselves for examination was successful, several of them showing a high degree of excellence. These are the first probationers who entered the infirmary for three years' training, and their success promises well for the future of the school. At Burton-on-Trent, Dr. W. G. Lowe reports to the Guardians that the three probationers whom he recently examined all passed in nursing and two in midwifery, the third only failing because illness prevented her from attending all the classes.

### A CO-OPERATIVE NURSING ORGANISATION.

An interesting meeting was held the other day at the instance of the Duchess of Norfolk in the great hall of Arundel Castle, the object being to discuss the possibility of promoting a nursing scheme for Arundel and district. A gratifying feature of the gathering was that Anglicans, Romanists, and Nonconformist ministers were all present, as well as medical men and a large number of ladies. The Duke of Norfolk presided, and called upon Mr. J. Bickersteth, of Hull, to explain the working of the Wilton Beacon Benefit Nursing Association in Yorkshire. This association is a co-operative organisation which enables all classes to secure the services of efficient nurses at a nominal expense. Twelve nurses are now employed by the committee, and there are now 1,200 subscribers of 2s. a year and upwards. The constant attendance of a nurse may be obtained for a weekly fee of 2s. 6d., 5s., or 10s., with board, according to the means of the members. Lady Margaret Bickersteth, the honorary secretary, stated that the association pays its way, and has encountered and overcome the religious difficulty. Eventually, after several of the company had expressed themselves in favour of the formation of a local association, it was agreed to leave the matter in the hands of the Duchess of Norfolk to take the preliminary steps.

### CROYDON GUARDIANS AND THE INFIRMARY CERTIFICATE.

The Croydon Guardians, we observe, are advertising in some of the Church papers for applications from educated young women, not under 22 years of age, for appointment and training as probationary nurses; and the applications are very properly to be addressed to the matron. But we think that until the signature of the matron is restored to the certificate of the Croydon Union Infirmary, there will be a difficulty in securing, even through the unusual medium of the Church papers, a sufficient number of probationers.

### THE FLORENCE SAUNDERS DISTRICT NURSING ASSOCIATION.

At a meeting of the Peterborough and District Nursing Association it was decided to change the title. The reason is that it is desired to inseparably connect with the Association the name of the late Miss Florence Saunders, who 18 years ago started the movement and for a long period acted as hon. superintendent. Dr. Walker, who read extracts from the will of Miss Saunders, according to which she leaves the Association £600 in cash, and a half share of a house valued at £1,200, stated that to his knowledge the superintendent had spent thousands of pounds in building up the Society. We agree with him that the Peterborough people can best show their appreciation of the generosity and devotion of Miss Saunders by subscribing the remainder of the sum which is required in order to enable the Association to acquire the house in which they already have a part interest if they choose. Moreover, Miss Saunders has left most of her furniture to the Association. In other words, she not only gave her money and her time without stint during her life to the work of nursing the sick poor, but her liberal bequest places it within the power of the committee to provide a central home for the district nurses of the future. Such a woman deserves to be always held in grateful remembrance in the city which is so deeply indebted to her.

### A NURSE FOR ROTTINGDEAN.

It has been decided to form a District Nursing Association for Rottingdean and neighbourhood, and a nurse will, accordingly, commence her duties on June 1st. She will live in the village, and act under the instructions of the committee. The rules of the Association provide that her services may be secured by payment of 4d. a month for any family whose head does not receive more than 25s. a week in wages; other persons paying from 5s. to 20s. These are not the conditions under which the Queen's nurses work, but the new Association is affiliated to the Sussex County Nursing Association.

### A HOME FOR FEVER NURSES.

The home which the committee of the Fever Hospital and House of Recovery, in Cork Street, Dublin, have been able to erect for the nursing staff attached to the institution, in consequence of a large legacy under the will of the late Mr. James Weir, has just been opened by the Lord Lieutenant of Ireland. It is a fine looking, handsomely laid-out building, has ample accommodation for the sisters, nurses, and probationers belonging to the hospital and engaged on the private staff, including separate sitting-rooms, dining-rooms, two writing-rooms, and 45 bedrooms. After Lord Dudley had received, and replied to, an address on behalf of the Managing Committee of the hospital, he was conducted through the building, and expressed himself highly pleased with the general arrangements which have been provided for the comfort of the nurses.

### SCHOOL PRINCIPALS AND HEALTH NURSES.

It appears that the public school principals and the nurses employed by the Board of Health in New York do not get on well together. The school principals do not always approve the methods of the nurses, and the nurses have declined to receive instructions from the school authority on the ground that they are responsible only to the Board of Health. The upshot of the matter is that the City superintendent has sent a letter to each of his school principals pointing out that the work of the nurses benefits not only the children and the community, but also the principals of schools, because the nurses, if given proper opportunity, can aid the principals in maintaining a high percentage of attendance. He therefore begs that the latter will co-operate with the nurses in the proper discharge of their duties. It would be a pity if an excellent movement were nipped in the bud by bad feeling or petty dissensions on either side.

### BERMONDSEY MEDICAL MISSION FOR WOMEN AND CHILDREN.

A feature in connection with the Bermondsey Medical Mission for Women and Children which has taken the place of the Church Missionary Medical Training Mission in Bermondsey, is that provision is made for training in medical, surgical, and district work. The length of training varies from three months to a year, and we are informed that lectures are given in elementary anatomy, physiology, medicine, surgery, midwifery, first aid, and home nursing. The practical work consists of surgical dressings at the dispensary every morning, maternity work in the homes of the people, and also district nursing. If specially desired, dispensing lessons can be given by a qualified dispenser. Of course, all this is only meant for ladies who wish to engage in an admirable philanthropic undertaking, and the Bermondsey Medical Mission does not enter into competition with nurse training schools.

### INCREASE OF SUBSCRIPTIONS AT BURSLEM.

At the annual meeting of the Burslem Nurses' Institution the announcement was made that there had been an increase in the subscriptions. This is all the more satisfactory because the income of the institution was £171 14s. 10d., as against expenditure £147 17s. In addition to the year's subscription £8 5s. was contributed to the special Nourishment Fund, and the committee were thus placed in a position to provide several needy patients with extra nourishment. We commend the formation of a special fund of this description to other organisations as one of great usefulness. It is intended to devote the interest from a legacy of £500 to the convalescent homes open to patients from Burslem at Llanfairfechan, Rhyl, Southport, and Buxton. This also is an excellent idea, and the subscribers to the society must be congratulated upon possessing a committee who seem fully equal to the discharge of their duties.

### PROGRESS AT CARLISLE.

Quite the feature of the report which was read at the annual meeting of the Carlisle District Nursing Association was the record of the help given to the work by special and extraneous efforts. A café chantant yielded £41 3s. 3d., a sale of work £24 16s. 9d., a fancy dress ball £9 13s. 6d., a lecture on radium £5 5s. This shows that the appeal which was made by the committee at the beginning of 1903 for further support did not fall on deaf ears; while another very encouraging fact is that the patients gave as thank-offerings £26 16s. 6d., an increase of more than £10 on the preceding twelve months. The final financial result was that the receipts amounted to £431 5s. 6d., and that after meeting all expenses, and paying £15 to the reserve fund, the committee had a balance in hand of £26 16s. 6d. Of the increase we notice that £206 only was received from subscriptions, and this should be materially increased, as the association cannot rely upon regularly receiving such large outside assistance as they enjoyed last year.

### FOOTBALL AND NURSING.

At the annual smoking concert of the Foleshill Football Club, a cup given by the Foleshill Nursing Association in recognition of the handsome contributions of the club to the expenses of the Association, was presented to Mr. H. J. Sharman, and a gold medal to Mr. A. F. Smith. The managers of the Association have given this practical proof of their belief in the principle of reciprocity, because they have enjoyed a record year in respect to receipts.

### DISTRICT NURSING AT HULL.

The progress of the Hull Jubilee District Nursing Association during last year was satisfactory from nearly every point of view. At the annual meeting, over which the Mayor presided, the report was presented by Mrs. Gore. The salient features are that during 1903 the nurses paid 31,304 visits, an increase of 3,894 over the previous year. One more permanent nurse has been added to the staff, so that there are now eight nurses at work in addition to the lady superintendent. Upwards of £40 was contributed by grateful patients in sums varying from 1s. to £3. This is a better way than levying a charge for services. Even with eight nurses, however, Dr. Nicholson says that Hull could do with twice the number; but though the association is generously supported by money, the balance is on the wrong side, and subscriptions will require to be considerably increased before the staff can be augmented.

### SHORT ITEMS.

Miss Florence M. Smith, the new nurse-matron of the Ancoats Hospital Convalescent Home, is at present not nurse-matron of Hayes Cottage Hospital, but Queen's nurse for Hayes district.—Miss W. G. Massey and Miss M. Worthington have been appointed staff nurses in Queen Alexandra's Imperial Military Nursing Service.

### The Nursing Outlook.

"From magnanimity, all fear above;
From nobler recompense, above applause,
Which owes to man's short outlook all its charm."

### FLORENCE NIGHTINGALE'S WORDS.

We are apt, in the wave of sentimentalism which surrounds Miss Nightingale's name, to remember only her sweetness and not her strength; only her praise and not her blame. We have come to regard her as one incapable of scorn, and yet the little black volume, "Notes on Nursing," that came out in 1860 contained some powerful denunciation and withering satire that is as deserved of the women of to-day as of the women of the past. Listen to this: "It seems a commonly received idea among men, and even among women themselves, that it requires nothing but a disappointment in love, the want of an object, a general disgust, or incapacity for other things, to turn a woman into a good nurse. This reminds one of the parish where a stupid old man was set to be schoolmaster because he was 'past keeping the pigs!'" So much for the nurse; and now let the patient have her rap over the knuckles in her turn. "There are two classes of patients which are unfortunately becoming more common every day, especially among women of the richer orders: (1) Those who make health an excuse for doing nothing, and at the same time allege that the being able to do nothing is their only grief; (2) those who have brought on themselves ill-health by over-pursuit of amusement, which they and their friends have unhappily called intellectual activity. I scarcely know a greater injury that can be inflicted than the advice too often given to the first class 'to vegetate,' or than the admiration too often bestowed on the latter class for 'pluck.'" Though this was written 40 years ago it is a true portrait of many patients of to-day; after all even Florence Nightingale cannot alter human nature, and we are none of us free from fault. And now one more example of vigorous rebuke that applies to all women. "If you like to wipe your dirty door, or some portion of your dirty wall, by hanging up your clean gown or shawl against it on a peg, this is one way certainly, and the most usual way, and generally the only way of cleaning either door or wall in a bedroom."

There can be no doubt about the acuteness of observation, the strength of mind, and the power of expression of the woman who wrote these terse phrases; they show us once more that it was not some mere mild damsel with gentle sympathies and tender heart who started the nursing movement in England, who organised the vast hospital at Scutari, who built the Nightingale Home at St. Thomas's, and who gave it model rules which all the world has copied. The public has always ignored Miss Nightingale's training in Harley Street, Kaiserswerth, and Paris; she burst suddenly on their vision as a risen star when she started for the Crimea; she was the flame, the fashion through the days of war; but, sadly enough, this same public has also ignored all the steady work she has done from her invalid couch in her old age. Certain Ministers of State might tell of Blue Books submitted to her and modelled on her advice; matrons can tell of advice sought and obtained, and movements forwarded and organisations founded. But we have all heard the wondering remark, "What! is Miss Nightingale still alive?" We have all seen the sentimental pictures of the lady draped in a shawl putting her hand on a patient's head. When she took her pen in her hand and wielded that for the improvement of tendence on the sick, there were few to hear, and seemingly few to heed. And yet in the long run this is the work that tells, the soil that lasts. Those terrible years in the Crimea, lived in the face of death, and in the fierce praise of the public, were shared with other women to whom the dangers to health were equally great, and whose spells of work were as hard and as long. But it was the mind within the woman, the keenness of eye, the greatness of heart, the mental courage as well as the physical courage. We all know good nurses who go through life doing their best with what they find, but never dreaming of trying to improve things, never daring to blame even open wrong. This was not Miss Nightingale's way: her reports to Lord Raglan were as brief and biting as some of the phrases we have quoted above; she was no more afraid to speak than afraid to do. Not that she ever used destructive criticism, but always constructive; she replaced the evil by the good. That is where the greatness came in—in the combination of sweetness and of strength, of gentleness and of power, of unselfishness and desire for the public weal. The South African war produced no Florence Nightingale. But the opportunity did not arise. Since the Crimean War the influence of Miss Nightingale has so revolutionised the position that nowadays it would be difficult for a star to arise. The darkness no longer exists. Many nurses went out to South Africa who, whether in respect to character or capacity, were all that could be desired—devoted, tactful, clever women. If there had not been a lamentable want of preparation and a deplorable lack of organisation, the proportion of nurses who did not answer this description would not have had the chance of reflecting discredit on their calling, and there would probably have been no cause for complaints that the high ideals even of Miss Nightingale had not been reached. In conclusion just one more quotation from the almost forgotten "Notes" to show truth enclosed in a trenchant phrase: "I have often been asked, When ought the windows to be opened? The answer is, When ought they to be shut?"

### The Modern Nursing of Consumption.

By Dr. Jane H. Walker, Physician to the New Hospital for Women, London, and Medical Superintendant of the East Anglian Sanatorium, Nayland, Suffolk.

OK

### LECTURE VI.

### Private Nursing of Consumption.

The nursing of consumptives in their own homes is a far more arduous task than in sanatoria. There is greater responsibility, and greater need for tact, judgment, and patience on the nurse's part. Very few patients, unless they be very ill, have or need a daily medical visit, and instead of every hour of the day, and almost every movement of the patient being regulated by the orders of the doctor in charge, the nurse will in most cases be called upon to settle minor details of food, rest, exercise, visitors' hours, etc., in accordance with the daily variations in her patient's pulse, temperature, and general condition. Upon her may largely depend the choice of a suitable room, the furniture, and the arrangements of many small domestic details, in all of which she will have to study her patient's welfare, and also his and his friends' idiosyncrasies. Unless he have already been at a sanatorium, she will have to educate her patient to living in the open air, indoors as well as out, to dissipate his fear of draughts and horror of the damp, and, more difficult than this perhaps, she will have to educate his friends, and to prevent them going in to shake their heads at the patient, looking, if not saying, that they are certain that he is on the high road to death or a lunatic asylum.

A nurse who is going to look after phthisis cases should certainly have a preliminary training in a well-managed sanatorium. In three to six months' training she will be able to appreciate fully the importance of the daily *régime*, and the unimportance of draughts, damp, rain, and even frost and snow. She will learn the best methods of keeping a patient comfortable while under the treatment without being coddled, and how to make him careful without being fussy. She will learn how to save the patient exertion and keep him cheerful without degenerating into a chronic and very dependent invalid. She will learn that it is food, and not the mixture three times a day, that is of real importance. She will learn that cases apparently hopeless may with time and care be not only benefited but cured, and above all, she will, or should, learn that the ordinary hospital routine, eminently suitable for acute or lingering medical cases, is not the form of nursing needed for a disease, which though very lingering is not hopeless, and where the sick individual is as much to be considered as the individual sickness.

*The Patient's Room.*—With regard to the selection of a room, subject, of course, to the doctor's approval and the exigencies of the case, the nurse should see that the patient has a room which faces a little west or east of south. East of south is better, as the S.W. wind is the prevailing one in this country, and an excess of wind is to be avoided, as it is apt to bring dust into the room. A windy room is also uncomfortable, and some think wind is even harmful for phthisis patients.

If the case be a bad one the room should be on the ground floor so that the bed or couch may readily be moved out into the open.

*Warming and Lighting.*—An ample supply of warm but light blankets and hot bottles must be provided. A patient can lie comfortably with doors and windows open during the coldest weather if his body be kept warm by a sufficiency of bedding. Except during dressing or bathing no heating of the room should be allowed. If the room is to be artificially heated of course the nurse must use the means provided, a fire is only to be permitted if no other method of warming be possible. In sanatoria steam or hot-water radiators are generally used, in private houses an open fire-place or gas stove is as a rule the only method of heating. A gas stove has the advantage of creating no dust, it should have a pan of water on the top or close by to prevent the air getting too dry. The best gas heating is asbestos laid in an ordinary fire-place, with the gas pipe running well at the back. Gas stoves are more apt to make the room gassy. Great care must be taken that there is no leakage in the joints of the gas pipes, and that the fire is not kept burning too continuously, or the heated and dried air will make the patient cough. If there be a fire the nurse must restrain her natural inclination to sit over it, for, besides being a bad example for the patient, she will feel the cold more when she moves away than she will if she resolutely hardens herself.

*Furniture.*—This should be of the simplest. No carpet must be allowed, a strip of matting or bath blanket (which can be easily washed) by the bedside is all that is needed. A small table by the bedside, a second one for books or flowers, a couch, a chest of drawers, small dressing-table and wash-stand are all the requisites that should be permitted. Patients' wearing apparel is much better kept out of the room, in some convenient and ventilated cupboard. If the furniture be of good design and look nice the room, unless it be very large, will not look bare. There is no object in having a patient in a very large room, a large, and especially a high room is much more difficult to keep really aired and clean. A room 20 feet by 14 by 10 is amply large enough for any patient, and one 10 feet by 14 by 8 will do extremely well.

It may be impossible to have anything on the walls except ordinary wall paper, but if practicable a washable distemper, such as Hall's distemper is preferable. Varnished paper should never be allowed, in cold and foggy weather moisture is precipitated on the walls, the room is constantly damp, and, even with the windows always open, will smell musty.

The floor should be beeswaxed and polished once a week, and rubbed daily with a damp duster. The nurse must see that the furniture is also dusted with a damp duster daily; this may ruin the polish, but no dry dust must be allowed to fly about in a patient's room.

*Windows.*—If these are of the sash variety the best plan is to take them out altogether; if of the French pattern, of course they can be kept always open, and of this kind those that open inwards are better than those opening outwards.

*Curtains.*—A thin curtain of linen or some washing material may be allowed if the sun disturb the patient much in the early mornings, and if the room be on the ground floor, but the patient will probably soon get used to a light room, and the curtains should be drawn as seldom as possible.

### To Nurses.

We invite contributions from any of our readers, and shall be glad to pay for "Notes on News from the Nursing World," or for articles describing nursing experiences at home or abroad dealing with any nursing question from an original point of view according to length. The minimum payment is 5s. Contributions on topical subjects are specially welcome. Notices of appointments, letters, entertainments, presentations, and deaths are not paid for, but we are always glad to receive them. All rejected manuscripts are returned in due course, and all payments for manuscripts used are made as early as possible after the beginning of each quarter.

### The Nurses of the East London Hospital for Children.

INTERVIEW WITH THE LADY SUPERINTENDENT: BY OUR COMMISSIONER.

It is not a legend that people unacquainted with East London have confessed to some difficulty in finding their way to the admirable institution which is so well known to tens of thousands of toilers in that part of the metropolis. But this need not be the case. Far down that great thoroughfare, Commercial Road, is Sutton Street, and here, on both sides of the street, are tablets with the words, "To the East London Hospital for Children," so conspicuously inscribed, that none with eyes to see can fail to observe them. The hospital cannot be missed if the Sutton Street route is taken, but a little further on in Commercial Road is Harding Street, and this leads straight to its doors in Glamis Road. Certainly, no one need be deterred from going to ascertain for themselves how the sick children of the East End slums are nursed by the idea that Shadwell is inaccessible.

On the occasion of my visit for the purpose of a talk with Miss A. Row, who has been lady superintendent for nine years, the sun was shining so brightly and the weather was so delightful that a large number of children, many in their beds, were enjoying the air in the pleasant yard outside. I inquired if a nurse was always with them, and the lady superintendent replied that nurses were constantly in and out. Moreover, as I observed for myself immediately, the nurses inside the ward looking on to the yard can see all that is going on. Directly the weather and the state of the patients permit, they are kept out of doors as much as possible. Both in the cheerful, well-ordered wards, gay with flowers, and out of doors, the great majority of the children seemed wonderfully happy and contented, though now and again a tiny form with wan face lying back on the pillow suggested serious illness, or a pathetic wail proclaimed the presence of pain. The youthfulness of the nurses is, of course, a feature, and one of the first questions I asked the lady superintendent was the age at which a probationer is admitted.

### Paying Probationers.

"That depends," she replied, "upon whether she is a paying or a non-paying probationer. If the former, she must not be under 20 nor over 30; if the latter, the age is 20 to 26."

"How many paying probationers do you take?"

"Five. They pay a guinea a week, and can stay for either six or twelve months. At the end of six, if they choose and are found eligible, they can become non-paying probationers for the remainder of the three years."

"The full period of training?"

"Yes; three years' training has been in force here for some years. Originally the period was two years, but I think three essential. The work in the wards is precisely the same for paying as it is for non-paying probationers."

"Do many of them go in for the full course?"

"A very fair proportion. Those who go at the end of six months or a year merely have a letter given them to the effect that they have been in the hospital for a certain time. Only a few, I think, come with the idea of not staying on, while many start as paying probationers with the hope of securing the earliest vacancy on the regular staff.

### The Regular Staff.

"How many probationers are there altogether?"

"Thirty, in varying stages of training. The salary of the ordinary probationers is £10 for the first year, £12 the second year, and £20 the third. Indoor uniform is given, but nurses who like to wear outdoor uniform, which is optional, have to provide it for themselves. We get plenty of applications."

"And the number of sisters?"

"Seven, with the home sister. Several of the sisters had their children's training here, and all have had three years' training at a general hospital. No one would be accepted as a sister who had not been trained in both a children's and a general hospital."

"What about lectures?"

"During the winter months a course is given by the resident medical officer to the senior probationers, and by one of the sisters to the juniors. The subjects of the lectures vary, and they are not of the same importance as at the general hospitals. At the termination of the three years the certificate is signed by the senior surgeon, the senior physician, the lady superintendent, the chairman of the governing body, and the secretary."

### Hours and Holidays.

"When do the probationers and staff nurses go on duty in the day?"

"At seven o'clock, and they are off duty at six, except the nurses who take evening duty between six and nine, but these have three hours off in the afternoon. Half an hour is allowed for lunch, also for tea, and three-quarters of an hour for dinner. They breakfast before they go into the wards."

"Then a considerable number get their evenings practically free?"

"For the three hours certainly. But all the nurses have also a night and a day off once a month, leaving at six in the evening and returning at ten the next evening. This gives them an opportunity to go to a theatre if they like. They have a month's holiday."

### The Sisters and Night Work.

"Then, I suppose, that the sisters have five or six weeks?"

"No, a calendar month. The sisters have a longer time off duty on Sundays, though the time off duty on Sundays for all depends upon the work that has to be done. The sisters are in the wards at 8 A.M., and leave three days a week at six, three at nine, and one at two. Their salaries begin at £30, and increase to £40."

"With regard to the staffing of the wards, how many nurses are attached to each?"

"The medical and surgical ward for boys, with 36 beds, and the medical ward for girls, with 31 beds, have six day nurses attached to them, and the surgical ward for girls, with 30 beds, five. I include in the figures for the boys' ward four beds in the adjacent ward. A sister is in charge of each ward, and there is one on duty at night in charge of the whole hospital."

"How many nurses are on duty during the night?"

"One staff nurse and one probationer in each ward. They come on duty at nine and are off at eight in the morning. During the night they each have one meal away from the ward, it being arranged that the ward is never left without a nurse in charge."

### The Out-Patients' Department.

"Then there is the isolation ward. It contains ten beds, but the accommodation is badly arranged, and as soon as the two wards of six beds each, for whooping-cough cases now in course of erection are completed, they will be used for the ordinary infectious cases while the existing isolation block is pulled down and reconstructed on modern lines.

"The out-patient's department is very large. Do you know how many it will hold?"

### THE NURSES OF THE EAST LONDON HOSPITAL FOR CHILDREN—*Continued.*

"Several hundreds. It is a most important branch, and there are always on duty a sister in charge, a staff nurse and two probationers."

"Judging by the appearance this afternoon the majority of the patients are babies."

"We admit children from the hour of birth up to 14. But the majority of both in and out-patients are certainly very much under that age, and are mainly babies. While the friends of the children are waiting their turn they can buy tea and a bun for a trifle in the department, which is open at nine and closes at six."

"The nurses on duty must have a good deal of valuable experience?"

"That is true, I think, of the whole hospital. In the out-patient department there are a good many dressings to do. One nurse is always in the room with the physician or surgeon who sees the case."

"Is there a nurse always on duty in the casualty department?"

"Up to 10 P.M. The department itself is always open. There is a sister in charge of the theatre, but she works single-handed."

### The Nurses' Quarters.

"You have no separate home for the nurses?"

"Only a house adjacent where six of the night nurses in charge of a sister are accommodated, with a maid to attend them. The day nurses sleep either in the cubicles at the top of the building or in rooms in other parts of the hospital. They have a sitting-room of their own, and they dine in the board-room."

"The board-room is used as the dining-room?"

"Yes. The sisters dine by themselves, and they have their own sitting-rooms off the wards."

"You would like to have more space available for the nurses?"

"There is space available, if we had the money to build. But the cubicles are perfectly airy and are as private as ordinary rooms."

### The Cases.

"Do you care to say anything about the cases?"

"There are a great many bad medical cases. The ailments vary from a burn to a case involving a most serious operation. Most of the cases come from the district round the hospital, though a few are sent from East and West Ham. But they are of the same class."

"The very poorest of all?"

"Yes, and therefore the most necessitous. Unfortunately, not only is the hospital always full of patients, but a great many children who ought to be in it are sent away because there are not beds for them."

"Is the Jewish element largely represented?"

"There are a good many Jewish patients, and we have children of almost every nationality, including Russians, Poles, and Italians. There is no difficulty with the patients in respect to language, they learn to speak English quickly. But we have a little trouble with the parents sometimes."

"I suppose that all religions are also represented?"

"They are. But the religious question is never raised at all here. One of the clergy in the neighbourhood is chaplain to the hospital, but we have no objection to Roman Catholic nurses; and intending probationers are not asked whether they are Churchwomen or Nonconformists."

"Do you get many infectious cases?"

"All things considered, no. But of course we are always liable to them in the wards. We do not take in any except very bad cases of diphtheria when an operation is required; and such cases go straight into the isolation ward."

### The Nursing of Children.

"Is it your opinion that children are more trying to nurse than adults?"

"In many ways they are, but we do not find that the children here give much trouble. Occasionally we have a child who cries in the night and keeps the other occupants of the ward awake, but you cannot expect absolute quietude in a building which has nearly a hundred sick children in it. Generally speaking, the children are easy to amuse and pacify. They get plenty of toys and books. These are usually sent at Christmas, when we have entertainments which are quite a feature, and are eagerly looked forward to by the inmates."

"Can you suggest any particularly indispensable qualities for the successful nursing of children?"

"A children's nurse has to be more observant in little things than a nurse attending on adults. There are so many things that can only be found out by carefully watching children. Patience also is urgently needed; no one who does not possess it in abundance should attempt to be a nurse in a children's hospital."

### The Value of Experience.

"There is one other point. So far as the experience of nurses in this hospital goes, do the general hospitals welcome or otherwise applicants for probationerships who have received training here?"

"I am afraid that some of the general hospitals object to previous training. The argument against it is that the health of young women who have been in training in an institution for three years is not likely to be so satisfactory as that of those who are fresh from home. But most of our nurses have a good long rest after their training before they begin work again."

"You do not, in fact, think that there is much force in the argument?"

"Very little. Against it may be set the advantage of the experience gained. That must help the nurses who enter as probationers in a general hospital. At any rate, they learn all the initial duties here, understand and appreciate the need of discipline, and know what hard work means."

"The work, though, is not so hard as in a general hospital?"

"On the contrary, it is harder, and this is the view of nurses from general hospitals who occasionally undertake holiday duty here. But a fairly robust girl will not find that it over-taxes her physical resources, and it has many compensations."

### TRAVEL NOTES AND QUERIES.

By our Travel Correspondent.

Brittany or Normandy (Sister Lilian).—You are quite in order and I am very pleased to help you, but I fear you want too much. There is no place near Dieppe where you could find accommodation for 5 francs a day, especially in August. Such terms can only be met with in out-of-the-way places, far from golf links and evening gaieties. The best I can suggest is St. Servan, where in August terms will be 7 francs per day. Write to Madame Pallot, Maison Mathias, St. Servan, Ille et Vilaine, and ask her for terms. You would be near enough to Dinard for golf and the casino. If you go there, the crossing is unfortunately rather long—Southampton to St. Malo, 10 hours. How would you like Caudebec on the Seine, about the same terms and within easy distance of Rouen? The neighbourhood of Dieppe and Puy is very dear. Write me again if I can help when you have further matured your plans. If you want a little mild gaiety I should rather suggest Scheveningen in Holland; it is well placed for excursions, but the country itself is flat and uninteresting.

### The Training and Supply of Midwives.

A MEETING of the Council of the Association for Promoting the Training and Supply of Midwives was held last week, by permission of Mrs. Charles Schwann, at 4 Princes Gardens.

The chair was taken by Miss Wilson, President of the Midwives' Institute and Member of the Central Midwives Board. Among those present were:—Lady Balfour of Burleigh, Lady Lucy Hicks-Beach, the Hon. Mrs. Egerton, Lady Norman, Lady Prideaux, Miss Rosalind Paget, Dr. Boxall, the Rev. T. H. Cavell, Mr. Charles Schwann, M.P., and Dr. Salaman. Among the new Members of Council elected were the Duchess of Montrose; Muriel, Viscountess Helmsley; the Countess of Lytton, and Lady Brassey.

### THE REPORT.

Mrs. Wallace Bruce, the Chairman of the Executive Committee, stated in her report that Mr. Arthur A. Leon, L.C.C., had consented to become Treasurer of the Association, and that Mr. Cosmo Bonsor (Treasurer of Guy's Hospital), Mr. Raffles Hughes, K.C., and Dr. Salaman, of the London Hospital, had joined the Finance Committee. She reported that a definite scheme of training had been drawn up, and in order to secure midwives for nursing among the poor, in town and country, free or assisted training was given by the Association to candidates willing to bind themselves to district midwifery for a definite period. Training vacancies had been engaged by the Committee at Queen Charlotte's Hospital; the Lying-in Hospital, York Road; the East End Mothers' Home; the Glasgow Maternity Hospital; and the Cheltenham District Nursing Home. Reference was made to the training home at East Ham in connection with the Plaistow Maternity Charity started by the Association last year. Since the opening of the Home in November, 1903, 189 infants had been under the care of the midwife-in-charge and the pupils under training, and more than 4,000 visits had been paid. As the work is in a very poor district an immense amount of good is thus done to many mothers and infants, and it has proved an excellent training-ground for the pupils. The cost of 16 weeks' training, with board and lodging, is £19 6s. 8d. The need of arousing local interest in the question of training midwives in accordance with the Act of 1902 is fully recognised by the Committee, and drawing-room meetings had been held at Hastings, Battle, Birkenhead, and Newport; while others are in course of arrangement. A meeting at Leicester convened by the National Union of Women Workers had been addressed by Mrs. Wallace Bruce, and a representative of the Association will speak in June at one of the meetings in connection with the Cartwright Memorial Exhibition recently opened at Bradford. It was stated that the question of finance was one of great anxiety to the Executive Committee. Sufficient funds were in hand to meet the expenses of the year, but there was no room for expansion. No further training could be undertaken than that already mentioned, which the Committee are well aware is but a very small measure of what is needed. They feel that a strong effort must be made to raise money. The sum of £1,000 would train 50 midwives, and it was felt that a direct appeal must be made for this amount to form the nucleus of a training fund. The Committee had been greatly encouraged by several generous donations; two of £20 each had been sent for special training, and it was felt that if more people would show their interest in this way the work would be greatly strengthened.

### THE DISCUSSION.

Some interesting discussion took place on various methods of getting into touch with local authorities and others interested in the question in view of the evident difficulties which must arise in providing midwives in sufficient numbers to meet future requirements; and it was resolved, on the motion of Lady Balfour of Burleigh, "That a large public meeting be held in the autumn." This was seconded by Dr. Salaman, who said he felt strongly that every effort should be made to supply an adequate number of midwives, for he considered that healthy infants were the mainstay of the nation and as needful as an efficient army.

In order to facilitate the business of the Association, a resolution was passed to the effect that the annual meeting be held early in the year instead of at Midsummer, as had been previously arranged. According to the constitution of the Association the financial year ends on December 31st, and it was considered more satisfactory that the working year should also end at that date. The annual meeting of subscribers and friends of the Association will therefore take place early in 1905. The office of the Association is at 12 Buckingham Street, Strand, where the secretary is in attendance from 10 to 1 o'clock daily, except on Saturdays.

### Everybody's Opinion.

[Correspondence on all subjects is invited, but we cannot in any way be responsible for the opinions expressed by our correspondents. No communication can be entertained if the name and address of the correspondent are not given as a guarantee of good faith, but not necessarily for publication. All correspondents should write on one side of the paper only.]

### PRINCESS MARY VILLAGE HOMES.

"A. G." writes: I have been matron at a large public-school sanatorium for several years. Always, in my absence, the "nurse in charge" sent or reported straight to the medical officer. I think the bursar, office clerk, or any "lay" official would have been rather surprised if she had applied to them; besides, in this case the nurse states that she was engaged as charge nurse entirely, subject, of course, to the matron's authority; it therefore seems very unfair that she was reprimanded for duty faithfully done according to the terms of her engagement.

### THE ROYAL NATIONAL PENSION FUND FOR NURSES.

"A NURSE" writes: Will you permit me, through the medium of your paper, to appeal to nurses who do not belong to the Royal National Pension Fund? I feel I cannot refrain from doing so. As one who has been a member of that Fund for 15 years, I would ask them to weigh earnestly the reason they are not members of it, and next to take the step of writing for details and asking questions. I can assure them that they will receive most helpful answers and advice, and it will be borne in on them what one has so deeply felt, that the nurses' truest and best welfare is the sole interest the Council has at heart. I cannot find words adequately to express all the kindness and help I have received, nor to convey my thanks. The thought that one is providing for one's future has been a great comfort and given a feeling of rightful independence.

### STATE REGISTRATION.

"NURSE EVA" writes: On reading in last week's HOSPITAL about State registration, the question arose in my mind, How will it affect me? Doubtless there are several nurses who are situated much the same as myself. It had been my ambition for years to become a nurse, and I looked forward to the time when I should be able to enter St. Thomas's, but for a while home duties held me. When left comparatively free, judge my disappointment when the doctor said he could not give a certificate for health. After a long rest I determined to try again, and although still unable to enter a general hospital, succeeded in being accepted on trial as probationer in a home for sick women. There I completed one year's training, after which I went to Queen Charlotte's Hospital, and got a certificate for monthly nursing. At present there is no difficulty in getting an appointment under the Poor Law. Will State registration make any difference? Can you suggest any plan by which such an one as myself by working in the meantime could become eligible for registration? My whole heart is in nursing the sick poor, and without my work life would be a blank.

### AN ALTERNATIVE TO REGISTRATION.

"BONNYGRASS" writes: Having read Miss Lückes' reply to Lady Helen Munro Fergusson's paper on State Registration for Nurses, and having heard my friends who favour the movement discuss the pros and cons, I suggest as an alternative (taking a leaf from our German neighbours) that each nurse on leaving the hospital where she has been trained, after gaining her certificate, be presented with a testimonial book from her training school, and that after each appointment held or case nursed, the book should be signed by the medical man and employer, testifying to her efficiency (a few words of approval or "damning with faint praise" would give the key to the woman's character). Having regard to the "personal element" that Miss Lückes emphasises so much, and which must always enter into the work, would this not prove a safeguard to the public against the half-trained nurse or the actual impostor? I just throw this out as a suggestion.

### A BAD CASE.

THE MATRON of a Benevolent Institution writes: Will you kindly give publicity to the following, as it may deter others displaying the same mistaken kindness? Some time ago I advertised for a district nurse. An applicant was chosen whose testimonials were very good, two being from the medical men, one from the secretary of an association. She had only been at work a few weeks when she betrayed habits of intemperance for which she had to be discharged. Upon making inquiries from the secretary of the association who gave the testimonials, the officials, alas! had experienced the same; yet, under the circumstances, gave testimonials, thereby endangering the lives of the poor women whose lives we try to brighten and help in times of sickness. I suppose that the original testimonials will be used to the detriment of the profession and the great danger of the patients. I feel strongly that there is too much sentiment in giving testimonials. One should be just to both sides.

### THE UNTRAINED MIDWIFE.

"NURSE H. J." writes: There is a great deal written in the present day about "The Midwives Act," "The Registration of Midwives," etc., and the sooner it is brought into force the better. I read in THE HOSPITAL lately of a so-called midwife (untrained of course) who washed a newborn infant in whisky and water and gave it a teaspoonful of castor oil! We will imagine such a nurse at a confinement case, should labour set in before medical assistance could be procured. How could she diagnose whether the case was normal or abnormal? What would she know about the use of antiseptics, so essential in the puerperal state. How would she deal with a post-partum hæmorrhage or the various complications which may always occur in a mismanaged labour? Think of the danger to the mother. What does she know about the baby? How many a child is blind from inattention to its eyes at birth? Surely such a state of affairs is wrong, and it will be an immense advantage when the provisions of the Act are enforced.

### Appointments.

GREEK HOSPITAL, ALEXANDRIA.—Miss Emma Davis and Miss Alice Iremonger have been appointed sisters. Miss Davis was trained for general nursing and sick cookery at the Poplar and Stepney Sick Asylum, and for fever training at the Eastern Hospital, Homerton. She has since her training been staff nurse at the Poplar and Stepney Sick Asylum. Miss Iremonger was trained for general nursing and sick cookery at the Poplar and Stepney Sick Asylum, and for fever nursing at the Fountain Hospital, Lower Tooting. She has since her training been staff nurse at the Poplar and Stepney Sick Asylum.

ROYAL NATIONAL HOSPITAL FOR CONSUMPTION FOR IRELAND.—Miss Violet Wyatt (*née* Müller) has been appointed lady superintendent. She was trained at the Western Infirmary, Glasgow. She has since been sister of out-patients' department at the Royal Hospital for Sick Children, Edinburgh, sister-in-charge of the Huntingdon County Hospital, matron of the District Hospital, Bushey Heath, Herts, and sister-in-charge of Pavilion I. at the Consumption Sanatorium of Scotland, Bridge of Weir.

ST. MICHAEL'S ORPHANAGE, WOODSIDE, CROYDON.—Miss F. E. Caper has been appointed matron. She was trained at St. George's Infirmary, Fulham Road, London, and has since been charge nurse at Sutton Schools, assistant matron at Ladywell, and matron of St. Olave's School, Sutton.

STAMFORD, RUTLAND, AND GENERAL INFIRMARY.—Miss Julia Vaughan has been appointed matron. She was trained at Peterborough Infirmary, where she has since been head nurse. She has also been charge nurse at the Royal Hospital, Richmond, Surrey.

### Novelties for Nurses.

BY OUR SHOPPING CORRESPONDENT.

### THE "STORK" HOSPITAL SHEETING.

FOR the "Stork" hospital sheeting I anticipate a great demand. Until recently the only waterproof materials were oiled silk, or cotton, and rubber. The disadvantages of rubber are well known, while oiled fabric has often proved itself to be a very broken reed. "Stork" sheeting seems to supply a want long felt by doctors and nurses. While possessing none of the undesirable characteristics of mackintosh, it is absolutely waterproof and antiseptic. It is made in two thicknesses, light and heavy, and is white, soft, and pliable. The light is an almost ideal make of waterproof. I have sewn it easily—with an ordinary fine needle and cotton—and proved that it will hold even boiling water. For use in the nursery, babies' cradles and carriages, nurses' aprons, sleeve protectors, etc., it could hardly be equalled. The manufacturers also supply "Stork pants" for babies, made of the same thin material—admirable little garments for superseding the old waterproof pilch. The heavy "Stork" sheeting is made on a thicker material, and is specially adapted for obstetrical cases, or wherever a more substantial fabric is desired. Nurses will appreciate the difference in scrubbing and handling this sheeting compared with the toil of cleansing and lifting ordinary cumbersome mackintoshes. Both materials wash easily and do not get hard or stiff. The price of the "Stork" sheeting is very moderate; it is manufactured only by The Hospital Sheeting Company, 74 Broad Street, Boston, Mass., and can be obtained from their London depôt, 85 Fore Street, E.C.; and from their agents, Messrs. E. and R. Garrould, Edgware Road, London, W.

### Echoes from the Outside World.

### Movements of Royalty.

The King and Queen, accompanied by the Prince and Princess of Wales and Princess Victoria, left London for Windsor on Saturday afternoon. In spite of the drizzling rain, a number of spectators assembled near Buckingham Palace gates to witness their departure. The Prince and Princess of Wales drove from Marlborough House to Paddington. On the arrival of the Royal party at Windsor Castle, the Palace guard, mounted near Henry VIII.'s gate, saluted them as they ascended the hill. On Saturday evening his Majesty gave a dinner party, and on Sunday morning the Royal party attended Divine service in the private chapel. By the King's command, the bands of the Royal Horse Guards and the Coldstream Guards played on Sunday afternoon on the East Terrace, and several thousand residents and visitors were admitted to the promenade and garden. On Monday the King went to Hurst Park in his motor car, and the Queen drove out in the grounds at Windsor.

### The Alake of Abeokuta.

Our latest distinguished visitor is the Alake of Abeokuta, who arrived with the Prince Ladapo Ademola and his small suite on Sunday evening at Plymouth. They were met by Sir William McGregor, Governor of Lagos, who accompanied the party to London. The Alake's kingdom is a great tract of rich land in the British Protectorate of Lagos. The capital, Abeokuta, is a thriving city with a population of 70,000, distant 60 miles from the coast. The Alake himself is said to be the most progressive of West African potentates. He has become a Christian, and is establishing schools throughout his country. During his stay here the Alake will be received by the King, and he is to visit the cotton manufacturing districts in the United Kingdom, in which, as he grows cotton, he is deeply interested. On Monday he remained at his hotel, but on Tuesday he paid an informal call at the Colonial Office, drove to Madame Tussaud's, and was present at the performance at the Hippodrome.

### The War in the Far East.

On Friday last Admiral Togo reported the loss, near Port Arthur, of the cruiser *Yoshino*, and the battleship *Hatsuse*. The former was rammed in a dense fog on the previous Sunday by the cruiser *Kasuga*, and the latter struck a Russian mine and sank. Ninety of the crew of the *Yoshino*, and 300 of that of the *Hatsuse*, were saved. According to a St. Petersburg telegram, the mines were laid by the Russians in imitation of the methods by which the Japanese succeeded in destroying the *Petropavlovsk*. A portion of the Japanese Yalu army has retired on Feng-hung-cheng. A force numbering 20,000 came upon 32,000 Russians posted in a strong position. They did not think it wise to risk a battle, and retreated in good order and with great rapidity. From Russian headquarters at Mukden it is announced that the Japanese main body, the strength of which is estimated at 80,000 men, remains south of the Russian force covering Liao-yang. Speaking in Devonshire on Monday evening, Sir William Walrond, M.P., Chancellor of the Duchy of Lancaster, said that the mines laid by the Russians outside their ports were a menace to the shipping of all nations.

### Death of a Bombay Philanthropist.

There died at Nauheim on Thursday last week, aged 65, the millionaire merchant and philanthropist of Bombay, Mr. Jamsetjee Nusserwanji Tata. He was the pioneer of the movement for the acclimatisation of Egyptian cotton in India, a movement which has been one of the factors contributing to the remarkable success of his group of mills, despite a series of crises in the cotton-manufacturing industry of the country. Mr. Tata also contributed to the industrial expansion of the country of his birth by the extension of the use of artesian wells and the introduction of cold storage. As to his philanthropy, by means of his scholarships, tenable by Indian youths of special promise in this country as well as on the Continent and in America, he afforded many of his younger fellow-countrymen exceptional opportunities for gaining technical knowledge; and the Indian University of Research, to be created at Bungalore as the outcome of his offer of an endowment of £200,000, will be the monument of his beneficent career. The funeral took place in the private chapel of the Parsee cemetery at Brookwood on Tuesday.

### The Origin of a Great Movement.

At a dinner in aid of the funds of the National Society for the Prevention of Cruelty to Children last week, presided over by Lord Roberts, an interesting statement was made by Mr. Choate, the American Minister. He said that it was now nearly 35 years ago since a kindred society was founded in New York. That society had its origin in an incident of which he had personal knowledge. A young lady had her attention directed to a case of cruelty in the house of one of her neighbours, and appealed to Mr. Henry Bergh, the president of the American Society for the Prevention of Cruelty to Animals, who helped her to obtain a warrant for the production of the child and the woman who had her in charge. The publicity given to the case called public attention to this class of crime, and the result was that a new society—the first of its kind—was created—namely, the Society for the Prevention of Cruelty to Children of New York. Mr. Choate said that the amount of harm which it had prevented could never be measured. A precedent was established, and the example had spread to other countries—to England, France, Italy, Spain, Germany, and indeed to all Christian lands. Thus it came about that the energetic sympathy and determined purpose of a single young woman had been the one touch of nature which proved the whole world kin.

### The Whitsuntide Holidays.

The unexpected beauty of the weather on Monday caused holiday-makers, who from the forecast had expected a dull cold rainy day, to take unusual advantage of the leisure at their disposal. Never have larger numbers been seen at the many country resorts around London; it is reckoned that there were no fewer than half a million of people in the Thames Valley, nearly 80,000 visited the Earl's Court Exhibition, a large proportion of them trying the new experience of the Hiram flying machine; 51,000 went to the Crystal Palace where there were many indoor attractions, as well as the charming grounds, which are now at their best. Amongst the items announced was a performance by the band of the Coldstream Guards. But after the arrangements had been made the King "commanded" them to Windsor. Learning, however, in the course of the afternoon, of their previous engagement, the King at once sent instructions that Lieutenant Rogan was to take a special train back to Waterloo to allow of their arrival at Sydenham in good time. Naturally, when these circumstances were explained to the vast audience, the band was greeted upon its appearance with vociferous cheers. At the Zoological Gardens the new kangaroo cage, where the animals have a paddock with miniature hills and ravines to run about in and can be seen jumping along as in their native state, was greatly admired; as was also the new aviary, a forest in miniature, where many birds are already nesting.

### Notes and Queries.

**FOR REGULATIONS SEE PAGE 12.**

*St. Bartholomew's Hospital.*

(61) I see that candidates as probationers at St. Bartholomew's Hospital are required to pass a preliminary examination. Will you kindly tell me what are the approved text-books, and if the examination is to be passed at the beginning or at the end of the month's preliminary trial?—*M. E. D.*

Send a stamped and addressed envelope to the Matron, asking for full particulars.

*Additional Training.*

(62) A nurse, with two years' hospital training, would like to know of other hospitals which give another year's training and certificate at the end of that time.—*M. A. B.*

The West End Hospital for Diseases of the Nervous System, 73 Welbeck Street, W., trains for one year. Practical application of massage and electricity are taught.

*Nursing Fees.*

(63) Will you kindly tell me what fee I could charge a doctor for nursing any of his family? He has always been most kind, and I should like to do something in return.—*Grateful.*

You must be guided by what you can afford. From your letter it seems that a guinea weekly would be a suitable fee.

*Return Fare.*

(64) Having made arrangements for my employers to pay my travelling expenses, can I claim my return fare if I leave on my own account?—*R. R.*

Not unless a stipulation to that effect was made when accepting the case.

*Homes for Nurses.*

(65) I should be glad to know of any home for nurses, as my health gave way about six months ago. I can afford to pay a small fee.—*M. W.*

Sir Julian Goldsmid's Home of Rest for Nurses, 12 Sussex Square, Brighton. The Memorial Institute, Almondsbury, near Bristol. The Royal West of England Sanatorium, Weston-super-Mare. For convalescent homes consult "Burdett's Hospitals and Charities;" for residential homes, see the "Englishwoman's Year Book."

*Abroad.*

(66) I am very anxious to go abroad; will you therefore very kindly give me the addresses of the Civilian Hospitals in Calcutta, Bombay, and Ceylon; of the Colonial Nursing Association, and of nursing institutions in Algiers.—*F. E.*

See lists of hospitals in India in "Burdett's Hospitals and Charities;" but your purpose would be better served, possibly, by writing to the Up-Country Nursing Association for Europeans in India, the address is, Mrs. Sheppard, 10 Chester Place, Regent's Park, N.W. The address of the Colonial Nursing Association is the Imperial Institute, S.W., and that of the British Cottage Hospital in Algiers, is Alger Mustapha.

*Paris.*

(67) I should be glad to hear of an association or agency for English nurses in Paris.—*Nurse T.*

We do not know of one.

*Masseuses.*

(68) Will you kindly tell me where there is an opening for a certificated lady masseuse?—*Inquirer.*

The Secretary of the Incorporated Society of Trained Masseuses, 12 Buckingham Street, Strand, W.C., might be able to advise you if you write.

**Handbooks for Nurses.**

"Nursing Profession: How and Where to Train." 2s. net.; 2s. 4d. post free.

"Nurses' Dictionary of Medical Terms and Nursing Treatment. By Honnor Morten. 2s. post free.

"A Handbook for Nurses." By J. K. Watson, M.D. 5s. net; 5s. 4d. post free.

"Practical Guide to Surgical Bandaging and Dressings." By Wm. Johnson Smith, F.R.C.S. 2s. post free.

"The Examination of Urine." By J. K. Watson, M.D. 1s. net; 1s. 1d. post free.

"Case-book for Private Nursing." 3d. net; 4½d. post free per copy; or per doz. 3s., post free.

The above works are obtainable of all booksellers, or direct from The Scientific Press, Limited, 28 & 29 Southampton Street, Strand, London, W.C.

### For Reading to the Sick.

### THE POWER OF PRAYER.

Thrice blest, whose lives are faithful Prayers,
Whose lives in higher love endure!
What souls possess themselves so pure?
Or is there blessedness like theirs?

*Tennyson.*

Lord, what a change within us one short hour,
Spent in Thy Presence, will prevail to make;
What heavy burdens from our bosoms take,
What parchèd grounds refresh, as with a shower!
We kneel! and all around us seems to lower;
We rise! and all—the distant and the near—
Stands forth in sunny outline, brave and clear!

*Trench.*

There are some who give up their prayers because they have so little feeling in their prayers—so little warmth of feeling. But who told us that feeling was to be a test of prayer? The work of prayer is a far too noble and necessary work to be laid aside for any lack of feeling. Press on, you who are dry and cold in your prayers, press on as a work and as a duty, and the Holy Spirit will, in His good time, refresh your prayers Himself.—*Bishop Ingram.*

Let your heart and desires continually hold converse with God, in heartfelt simplicity. Reflect on Him with feelings of love and reverence, and often offer up your heart, with all that you have and are, to Him, in spirit and in truth, as cordially and sincerely as possible. If through weakness or unfaithfulness you forsake this exercise, which is so incredibly helpful and beautiful, all you have to do is, meekly and heartily to begin again. . . . A little patience and courage alone are needed.—*Gerhard Tersteegen.*

There is a lesson to be taken to heart by those who are sick and unable to work. Success is not everything; it may come or it may not; let us only rest assured that no prayer, no honest work or endeavour carried out for God's glory with a pure intention, ever really fails of effect and of success, though we see it not. Let our part be to do the will of Him that sent us, be that what it may, working on, suffering quietly for the name of Jesus. This should be a comfort and instruction for the sick, who imagine they can do little for the souls so dear to our Lord and fain would do so much.—*Anon.*

If my feeble prayer can reach Thee,
O, my Saviour! I beseech Thee,
Even as Thou hast died for me,
More sincerely
Let me follow where Thou leadest!
Let me, bleeding as Thou bleedest,
Die, if dying I may give
Life to one who asks to live,—
And more nearly
Dying thus, resemble thee!

*Longfellow.*

